# Practical Notes on Diagnosis and Treatment

**Published:** 1910-06-11

**Authors:** 


### Practical Notes on Diagnosis and Treatment.

**Tertiary Syphilis.**

Medicinal treatment in many cases is only successful when the patient is put on a generous diet. If this fails, a continuance of the treatment should be tried at the seaside.

**Tonsilitis and Diphtheria.**

The exudate in follicular tonsilitis may coalesce into a definite pellicle, but even so it only rarely extends beyond the tonsil. It may encroach occasionally on the back of the pharynx, but never extends to the palate.—*Dr. Foord Caiger.*

**Foreign Bodies under the Nails.**

If it is found impossible to obtain any hold on the body after cutting the nail down, one should soften the nail over the foreign body with a 10 per cent. solution of caustic potash, and then scrape away the softened portion till the body is exposed.

**Boric Acid in Food.**

There is no doubt that the continued ingestion of even quite moderate amounts of boric acid in foodstuffs is capable of causing in many people considerable impairment of health, especially gastro-intestinal disturbances and headaches.—*Dr. Wiley.*

**The Phthisical Pulse.**

A rapid pulse occurring apart from pyrexia is frequently one of the earliest signs of phthisis. Irritability of the pulse is another fact in early phthisis. Anything which naturally quickens the pulse does so to an exaggerated degree in the early stages of tuberculosis.

**Salicylates in Rheumatism.**

Dr. Lees advocates large doses always given with double the quantity of sodium bicarbonate. Unless this is done there is risk of acid intoxication and of a condition resembling diabetic coma. The dose may be gradually increased till in an adult 450 grains in 24 hours have been given.

**Voice Strain.**

Careful and distinct articulation takes the strain so much off the larynx that all regular voice users should be careful to practise it.

**Tobacco Amblyopia.**

When the symptoms in a case of tobacco amblyopia have not persisted for more than three months, recovery is usually complete, provided that absolute abstention from tobacco is practised for at least twelve months.

**Itching in Scarlet Fever.**

This is sometimes very distressing; as a remedy nothing is more useful than sponging the body with a warm solution of sodium carbonate (gr. x. to ℥j.), to which a little mucilage has been added.—*Dr. Seymour Taylor.*

**Subphrenic Abscess.**

It has been said that the heart's apex is not displaced by a subphrenic abscess. But this is not universally correct. We can only say that in some instances it is considerably displaced, whilst in others it is not displaced at all."—*Dr. Gee.*

**Spectacles for Children.**

The apprehension that the wearing of spectacles by young people during play is fraught with danger is much exaggerated. Damage to the eyes from breakage of the glasses is exceedingly rare. I have met with no example of such an accident.—*Professor McHardy.*

**"Stitch in the Side."**

This is due to desiccation of the serous membrane, not necessarily of the pleura, the peritoneum being sometimes involved, but always in the region of the diaphragm, and appears often to be associated with turgescence of the liver or spleen during digestion, and so is usually brought on by exertion after a meal.—*Dr. W. Essex Wynter.*

